# Bacteriophage Therapy on an In Vitro Wound Model and Synergistic Effects in Combination with Beta-Lactam Antibiotics

**DOI:** 10.3390/antibiotics13090800

**Published:** 2024-08-24

**Authors:** Guillermo Santamaría-Corral, John Jairo Aguilera-Correa, Jaime Esteban, Meritxell García-Quintanilla

**Affiliations:** 1 Clinical Microbiology Department, IIS-Fundación Jiménez Díaz, Universidad Autónoma de Madrid, 28040 Madrid, Spain; guillermo.santamaria@quironsalud.es (G.S.-C.); john.aguilera@fjd.es (J.J.A.-C.); jesteban@fjd.es (J.E.); 2 CIBERINFEC—Consorcio Centro de Investigación Biomédica en Red (CIBER) de Enfermedades Infecciosas, 28029 Madrid, Spain

**Keywords:** bacteriophage, *Pseudomonas aeruginosa*, multi-drug resistance, beta-lactam, wound infection

## Abstract

One of the primary opportunistic pathogens that can cause a wide range of diseases is *Pseudomonas aeruginosa*. This microorganism can become resistant to practically every antibacterial currently in use, including beta-lactam antibiotics. Its ability to proliferate as biofilm has been linked to, among other things, the failure of antimicrobial therapies. Due to a variety of virulence factors and host immune system modifications, *P. aeruginosa* is one of the most significant and common bacteria that colonize wounds and burns. A novel therapeutic option for treating these multidrug-resistant (MDR) bacterial infections is the combination of antibiotics and bacteriophages. This approach has been linked to improved biofilm penetration, a decreased selection of antibiotic and bacteriophage resistance, and an enhanced antibacterial impact. Combining the F1Pa bacteriophage and beta-lactam antibiotics reduced the viability of the mature biofilm of MDR *P. aeruginosa* strains and suppressed bacterial growth in vitro. F1Pa critically reduced the amount of biofilm that MDR *P. aeruginosa* clinical strains formed in the in vitro wound model. These findings highlight the bacteriophage F1Pa’s therapeutic potential as a prophylactic topical treatment against MDR pseudomonal infections in wounds and burns.

## 1. Introduction

*Pseudomonas aeruginosa* is a Gram-negative, non-fermentative bacillus widely found in aquatic settings. It is a significant opportunistic pathogen that can cause a wide variety of infections, including burn and wound infections. Chronic wound infections affect about 6.5 million people in the United States alone, putting a greater financial strain on the healthcare system and having serious economic repercussions estimated to be worth USD 25 billion yearly [1]. One of the primary microorganisms responsible for wound- and burn-related bacteremia in patients is *P. aeruginosa* [2,3]. It is one of the most prevalent pathogenic microorganisms (7.9%) in such infections [4], as observed in numerous studies conducted in Europe to date [5,6,7,8,9,10,11,12,13,14,15,16,17,18,19,20,21,22,23,24,25,26,27,28]. *P. aeruginosa* burn infections with and without bacteremia have death rates of 77% and 49%, respectively [29,30].

Because of its strong intrinsic resistance to many antibiotics such as aminoglycosides, beta-lactams, polymyxins, and quinolones due to a poorly permeable outer membrane and multiple transport systems, *P. aeruginosa* has a high rate of antibiotic resistance [31]. In addition to its innate resistance, *P. aeruginosa* can develop resistance to almost all antibiotics on the market [32]. This resistance occurs against a variety of antimicrobials, including aminoglycosides, beta-lactams, and fluoroquinolones [33]. Certain bacteria may be multi-resistant, meaning they may withstand the effects of three or more antibiotic classes [34]. Moreover, the fact that this bacterium can form biofilms [35]—a collection of bacteria encircled by a self-produced biomatrix [36]—and is inherently resistant to phagocytosis and a wide range of antimicrobial drugs makes the failure of antimicrobial therapies much more significant. Compared to bacteria in a planktonic state, biofilm-forming microorganisms are 1000 times more resistant to antibiotic treatment and can avoid host immune responses [37].

Due to the absence of appropriate and efficient medicines, phage therapy is one of the most promising strategies being investigated by researchers to inhibit multi-resistant *P. aeruginosa* bacteria. Viruses known as bacteriophages enter bacteria, grow there, and then lyse the bacteria to death [38]. Compared to conventional antibiotics, bacteriophage therapy has several advantages, including the capacities to target specific bacterial species, fight species that are resistant to antibiotics, multiply at the site of infection, and eliminate biofilms [35,38]. Phage treatments are less likely to cause systemic side effects than antibiotics because they are species-specific and solely target pathogenic bacteria, sparing innocuous commensal bacteria [38]. Nonetheless, as each antimicrobial agent used in monotherapy with a particular target promotes the establishment of antimicrobial resistance, combination therapies employing various antimicrobial agents are the most appropriate clinical approach.

Bacteriophage–antibiotic combination in vitro therapy against MDR *P. aeruginosa* has been demonstrated [39,40,41,42,43,44,45,46,47,48,49,50]. Moreover, in vivo experiments and case reports have shown good results with an increase of survival or a decrease of the bacteria count [51]. According to in vitro studies, the use of temperate bacteriophages combined with suboptimal concentrations of antibiotics can significantly decrease the population of *P. aeruginosa* [52]. However, lytic phages can function as adjuvants in combination with antibiotics by decreasing the minimum inhibitory concentrations (MIC) of those antibiotics, thereby enhancing the susceptibility to antibiotics that were previously ineffective as a treatment. The mechanism of action of the antibiotics used in conjunction with phages is a significant determinant of this phenomenon [53]. F1Pa can disperse *P. aeruginosa* biofilm, favoring the passage of biofilm to the planktonic state, in a way proportional to the concentration of the phage [54], allowing these bacteria dispersed by the presence of the bacteriophage to become more susceptible to beta-lactam antibiotics, which are not good antibiofilm antibiotics but good bactericidal antibiotics against planktonic bacteria. Due to bacteriophages spreading biofilm, as F1Pa, and beta-lactam antibiotics inhibiting bacterial cell wall formation, there has already been evidence of the synergistic efficacy of bacteriophages and antibiotics against *P. aeruginosa* [55,56].

The present study evaluates the potential preventative effect of a bacteriophage infecting *P. aeruginosa*, vB_PaeP-F1Pa (described previously by our group as containing lysogenic genes, this phage is not ready for clinical use but can be used for in vitro analysis [54]), using an in vitro wound model of infection and describes the activity of combinations of antibiotics and the bacteriophage against multi-drug-resistant *P. aeruginosa* clinical strains in both planktonic and biofilm conditions.

## 2. Results

### 2.1. In Vitro Wound-like Model

**Figure 1:**
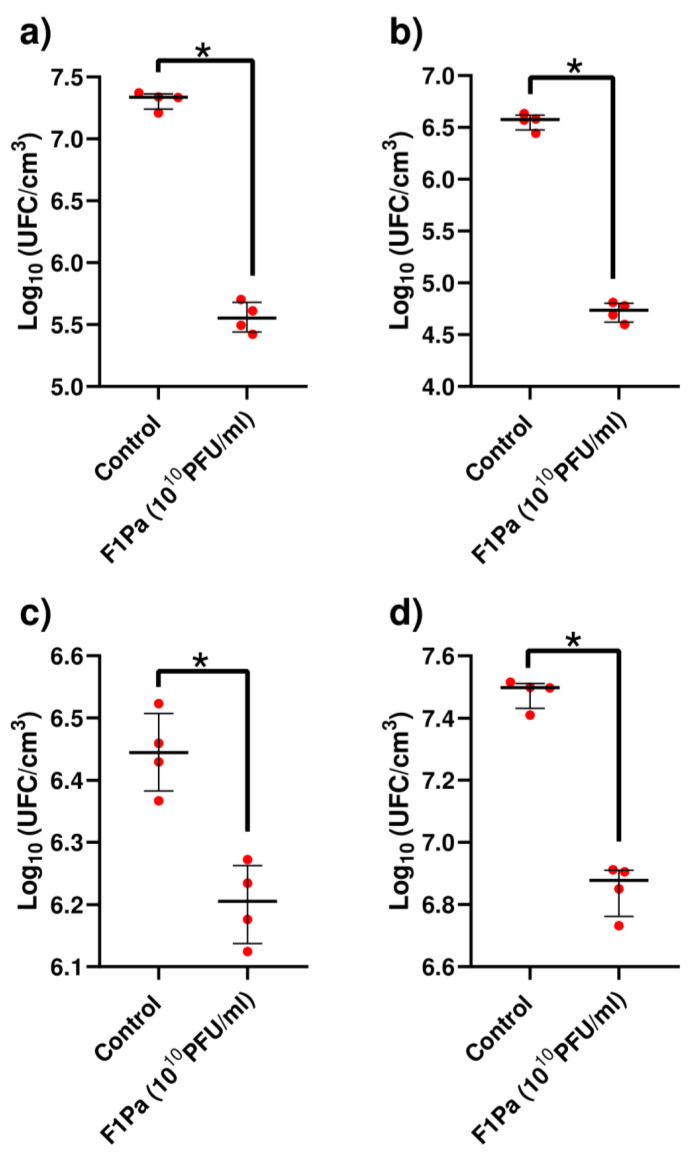
F1PA effect on wound biofilm formation of PAO1 (**a**), PA24 (**b**), PA35 (**c**), and PA36 (**d**) strains at 6 h. The bars represent the median and the interquartile range. *: *p*-value < 0.05.

The numbers of bacteria of PAO1, PA24, PA35, and PA36 associated with the wound biofilm of were reduced by 98%, 98%, 42%, and 76%, respectively, in the presence of 10^10^ PFU/mL F1Pa (*p*-value < 0.05) after 6 h of treatment (Figure 1).

### 2.2. Bacteriophage-Antibiotic Synergy

Combinations of carbapenems (doripenem (DOR), imipenem (IM), and meropenem (MP)), penicillins + beta-lactamase inhibitors (piperacillin/tazobactam (P/T)), and monobactams (aztreonam (AZ)) with the F1Pa bacteriophage were investigated on the PAO1 standard strain and the PA24, PA35, and PA36 clinical strains. These antimicrobial agents were used because they are beta-lactam antibiotics routinely used in the clinic against *P. aeruginosa* infections. For bacterial strains PA24 and PA35, the MIC of the bacteriophage was 10^9^ PFU/mL, while for PAO1 and PA36, the maximum concentration of F1Pa used did not inhibit bacterial growth (MIC(F1Pa) ≥ 10^10^ PFU/mL).

A synergistic effect was found between AZ, DOR, IM, MP, and P/T and the bacteriophage F1Pa for some of the strains. However, treatment of IM and P/T combined with F1Pa against the PA35 strain was not effective, and treatment of DOR with F1Pa displayed an additive effect. On the other hand, on the reference strain PAO1, treatment of AZ, DOR, and P/T with the bacteriophage showed and additive or synergistic effect (Table 1).

### 2.3. Phage–Antibiotic Inhibition Assays

**Figure 2:**
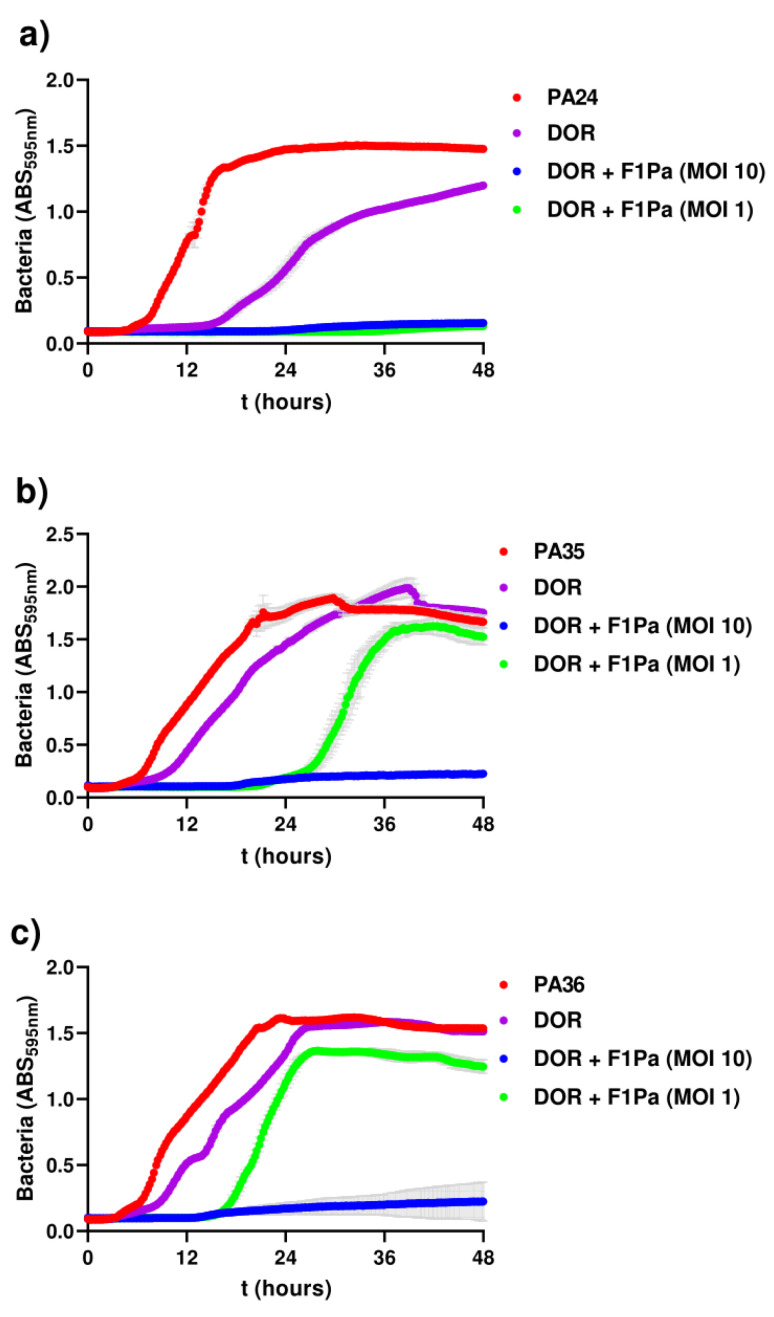
Inhibition of clinical isolates PA24 (**a**), PA35 (**b**), and PA36 (**c**) after treatment with F1Pa at different multiplicity of infection (MOI) values and combined with beta-lactam antibiotics. The grey bars represent the standard deviation.

The bacteriophage–antibiotic combination was examined through inhibition assays (Figure 2). The phage–antibiotic inhibition of clinical isolates (PA24, PA35, and PA36) was assessed for 48 h when infected with two different concentrations of F1Pa (MOI 10 and 1) and a single concentration of doripenem (8.79 µg/mL) (Figure 2a–c). An MOI 10 of combined treatments was able to inhibit completely the bacterial growth of the clinical strains tested in vitro, while a single treatment with a beta-lactam antibiotic was not capable of doing so.

### 2.4. Phage–Antibiotic Effect on Pseudomonal Biofilms

#### 2.4.1. F1Pa

**Figure 3:**
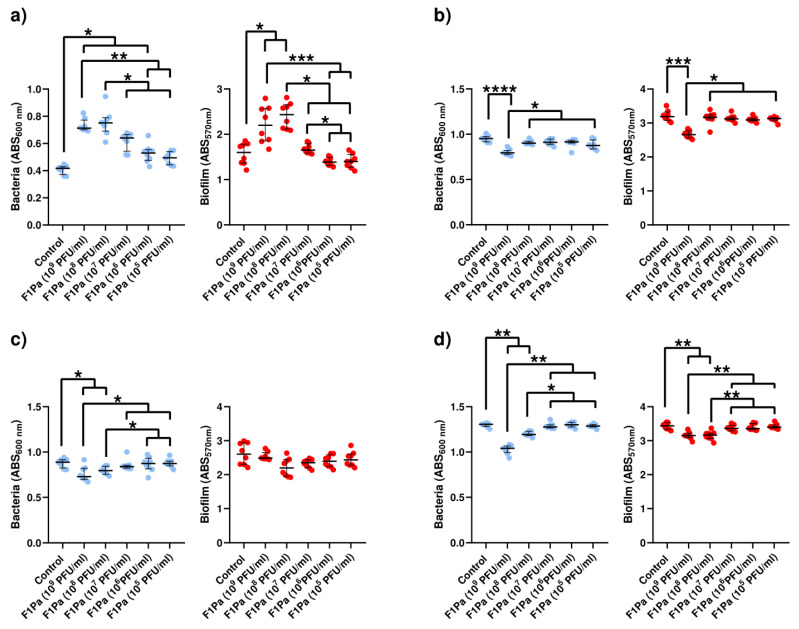
Effect of treatment with F1Pa on PAO1 (**a**), PA24 (**b**), PA35 (**c**), and PA36 (**d**) in both planktonic bacteria originating from the biofilm (**left**) and biofilm (**right**). The bars represent the median and the interquartile range. * *p*-value < 0.05, **: *p*-value < 0.01, ***: *p*-value < 0.001, ****: *p*-value < 0.0001 for Dunn’s test pairwise.

The concentration of PAO1 in planktonic bacteria derived from biofilm increased by 71%, 80%, 54%, and 27% in the presence of 10^9^, 10^8^, 10^7^, and 10^6^ PFU/mL F1Pa, respectively, (*p*-value < 0.01) at 24 h. The concentration of planktonic bacteria derived from biofilm and the concentration of the bacteriophage showed a very strong positive correlation (ρ = 0.9008, *p*-value < 0.0001) at 24 h. Concentrations of 10^9^ and 10^8^ PFU/mL F1Pa were able to increase the amount of PAO1 biofilm by 37% and 52%, respectively, (*p*-value < 0.01) at 24 h. The amount of PAO1 biofilm and the concentration of the bacteriophage showed a strong positive correlation (ρ = 0.6912, *p*-value < 0.0001) at 24 h (Figure 3a).

Only 10^9^ PFU/mL F1Pa was able to significantly reduce the concentration of PA24 clinical isolate planktonic bacteria derived from biofilm by 16% (*p*-value < 0.01) at 24 h. The concentration of planktonic bacteria derived from biofilm and the concentration of the bacteriophage showed a moderate negative correlation (ρ = −0.5221, *p*-value < 0.0001) at 24 h. In addition, only 10^9^ PFU/mL F1Pa was able to significantly reduce the amount of PA24 biofilm by 17% (*p*-value < 0.01) at 24 h. The amount of PA24 biofilm and the concentration of the bacteriophage showed a moderate negative correlation (ρ = −0.4319, *p*-value < 0.01) at 24 h (Figure 3b).

The concentration of PA35 clinical isolate planktonic bacteria derived from biofilm decreased by 18% and 10% in the presence of 10^9^ and 10^8^ PFU/mL F1Pa, respectively, (*p*-value < 0.05) at 24 h. The concentration of planktonic bacteria derived from biofilm and the concentration of the bacteriophage showed a moderate negative correlation (ρ = −0.5737, *p*-value < 0.0001) at 24 h. No concentration of F1Pa was able to reduce the amount of PA35 biofilm at 24 h. There was no correlation between the amount of PA35 biofilm and the F1PA concentration (*p*-value = 0.3871) at 24 h (Figure 3c).

The concentration of PA36 clinical isolate planktonic bacteria derived from biofilm decreased by 20% and 9% in the presence of 10^9^ and 10^8^ PFU/mL F1Pa, respectively, (*p*-value < 0.01) at 24 h. The concentration of planktonic bacteria derived from biofilm and the concentration of the bacteriophage showed a strong negative correlation (ρ = −0.7599, *p*-value < 0.0001) at 24 h. Concentrations of 10^9^ and 10^8^ PFU/mL F1Pa were able to significantly reduce the amount of PA36 biofilm by 8% and 8%, respectively, (*p*-value < 0.01) at 24 h. The amount of PA36 biofilm and the concentration of the bacteriophage showed a strong negative correlation (ρ = −0.7009, *p*-value < 0.0001) at 24 h (Figure 3d).

#### 2.4.2. Aztreonam-F1Pa

**Figure 4:**
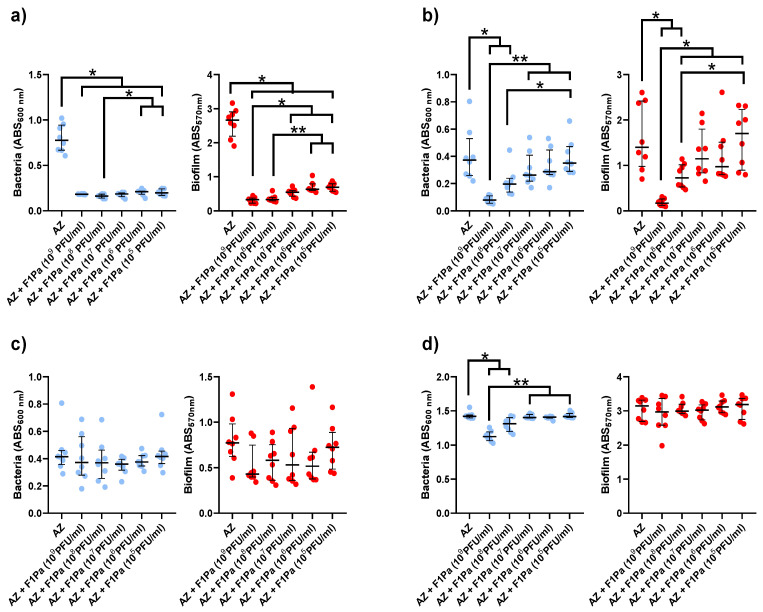
Effect of combined treatment with F1Pa and aztreonam (28.6 µg/mL) on PAO1 (**a**), PA24 (**b**), PA35 (**c**), and PA36 (**d**) in both planktonic bacteria originating from the biofilm (**left**) and biofilm (**right**). The bars represent the median and the interquartile range. * *p*-value < 0.05, **: *p*-value < 0.01 for Dunn’s test pairwise.

In the presence of any concentration of the bacteriophage mixed with AZ (28.6 µg/mL) (*p*-value < 0.05) at 24 h, the concentration of PAO1 planktonic bacteria sourced from biofilm dropped by 73%. There was a significant negative correlation (ρ = −0.6428, *p*-value < 0.0001) between the concentration of planktonic bacteria from the biofilm and the concentration of the bacteriophage mixed with AZ. When the bacteriophage and AZ were present in any concentration, the amount of PAO1 biofilm dropped by 74% (*p*-value < 0.05) demonstrating a strong, highly negative correlation (*p*-value < 0.0001, ρ = −0.8832). (Figure 4a).

The concentration of PA24 clinical isolate planktonic bacteria sourced from biofilm decreased by 79% and 47% in the presence of 10^9^ and 10^8^ PFU/mL F1Pa, respectively, combined with AZ (*p*-value < 0.05) with a strong negative correlation (ρ = −0.7159, *p*-value < 0.0001). Concentrations of 10^9^ and 10^8^ PFU/mL F1Pa combined with AZ were able to significantly reduce the amount of PA24 biofilm by 88% and 48%, respectively, (*p*-value < 0.05) exhibiting a strong negative correlation (ρ = −0.6833, *p*-value < 0.0001) (Figure 4b).

Curiously, no concentration of F1Pa combined with AZ was able to decrease the concentration of planktonic bacteria from PA35 clinical isolate biofilm, and there was no relationship between the planktonic bacteria from biofilm and the concentration of the bacteriophage (*p*-value = 0.1359). In addition, no concentration of the bacteriophage combined with AZ was able to reduce the amount of PA35 biofilm, showing a weak negative correlation (ρ = −0.3152, *p*-value < 0.05) (Figure 4c).

The concentration of PA36 clinical isolate planktonic bacteria from biofilm decreased by 22% and 10% in the presence of 10^9^ and 10^8^ PFU/mL F1Pa, respectively, combined with AZ (*p*-value < 0.05). The concentration of planktonic bacteria arising from biofilm and the concentration of the bacteriophage combined with AZ showed a strong negative correlation (ρ = −0.6719, *p*-value < 0.0001). However, no concentration of the bacteriophage combined with AZ was able to reduce the amount of PA36 biofilm. There was no correlation between the amount of PA36 biofilm and the concentration of the bacteriophage combined with AZ (*p*-value = 0.3013) (Figure 4d).

#### 2.4.3. Doripenem–F1Pa

##### Doripenem (8.79 µg/mL)–F1Pa

**Figure 5:**
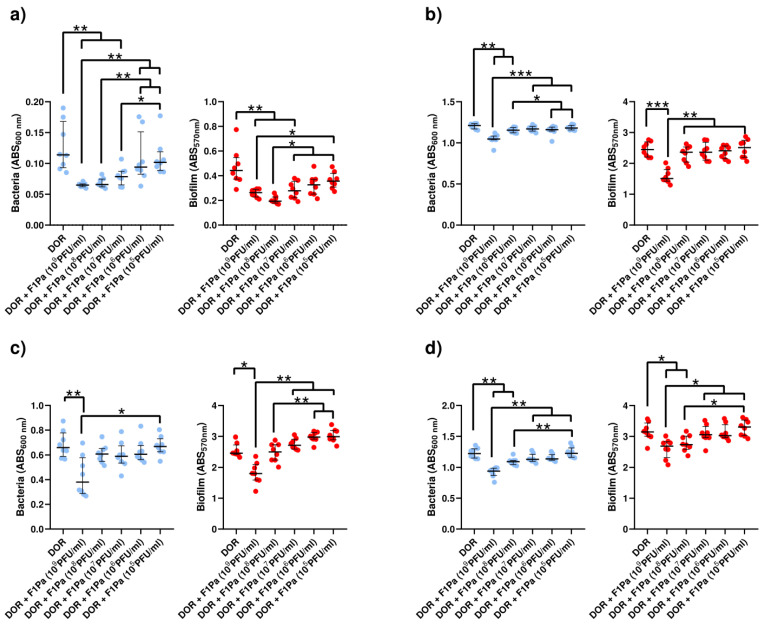
Effect of combined treatment with F1Pa and doripenem (8.79 µg/mL) on PAO1 (**a**), PA24 (**b**), PA35 (**c**), and PA36 (**d**) in both planktonic bacteria originating from the biofilm (**left**) and biofilm (**right**). The bars represent the median and the interquartile range. * *p*-value < 0.05, **: *p*-value < 0.01, ***: *p*-value < 0.001 for Dunn’s test pairwise.

The concentration of PAO1 planktonic bacteria derived from biofilm was decreased by 43%, 42%, and 31% in the presence of 10^9^, 10^8^, and 10^7^ PFU/mL F1Pa, respectively, combined with DOR (8.79 µg/mL) (*p*-value < 0.01) at 24 h. The concentration of planktonic bacteria derived from biofilm and the concentration of the bacteriophage combined with DOR showed a strong negative correlation (ρ = −0.7776, *p*-value < 0.0001) at 24 h. Concentrations of 10^9^, 10^8^, and 10^7^ PFU/mL F1Pa combined with DOR were able to reduce the amount of PAO1 biofilm by 40%, 56%, and 37% respectively, combined with DOR (*p*-value < 0.01) at 24 h. The amount of biofilm and the concentration of the bacteriophage combined with DOR showed a strong negative correlation (ρ = −0.7036, *p*-value < 0.0001) at 24 h (Figure 5a).

The concentration of PA24 planktonic bacteria derived from biofilm was decreased by 14% and 5% in the presence of 10^9^ and 10^8^ PFU/mL F1Pa, respectively, combined with DOR (*p*-value < 0.05) at 24 h. The concentration of planktonic bacteria derived from biofilm and the concentration of the bacteriophage combined with DOR showed a strong negative correlation (ρ = −0.6516, *p*-value < 0.0001) at 24 h. Only 10^9^ PFU/mL F1Pa combined with DOR was able to reduce the amount of PA24 biofilm by 38% (*p*-value < 0.001) at 24 h. The amount of biofilm and the concentration of the bacteriophage combined with DOR showed a moderate negative correlation (ρ = −0.5415, *p*-value < 0.0001) at 24 h (Figure 5b).

Only 10^9^ PFU/mL F1Pa combined with DOR was able to decrease the concentration of PA35 clinical isolate planktonic bacteria derived from biofilm by 42% (*p*-value < 0.01) at 24 h. The concentration of planktonic bacteria derived from biofilm and the concentration of the bacteriophage combined with DOR showed a moderate negative correlation (ρ = −0.5055, *p*-value < 0.0001) at 24 h. Furthermore, only 10^9^ PFU/mL F1Pa combined with DOR was able to reduce the amount of PA35 biofilm by 27% (*p*-value < 0.05) at 24 h. The amount of biofilm and the concentration of the bacteriophage combined with DOR showed a moderate negative correlation (ρ = −0.5125, *p*-value < 0.0001) at 24 h (Figure 5c).

The concentration of PA36 planktonic bacteria derived from biofilm decreased by 23% and 11% in the presence of 10^9^ and 10^8^ PFU/mL F1Pa, respectively, combined with DOR (*p*-value < 0.01) at 24 h. The concentration of planktonic bacteria derived from biofilm and the concentration of the bacteriophage combined with DOR showed a very strong negative correlation (ρ = −0.8004, *p*-value < 0.0001) at 24 h. Concentrations of 10^9^ and 10^8^ PFU/mL F1Pa combined with DOR were able to reduce the amount of PA36 biofilm by 14% and 13%, respectively, (*p*-value < 0.05) at 24 h. The amount of biofilm and the concentration of the bacteriophage combined with DOR showed a strong negative correlation (ρ = −0.6041, *p*-value < 0.0001) at 24 h (Figure 5d).

##### Doripenem (23 µg/mL)-F1Pa

**Figure 6:**
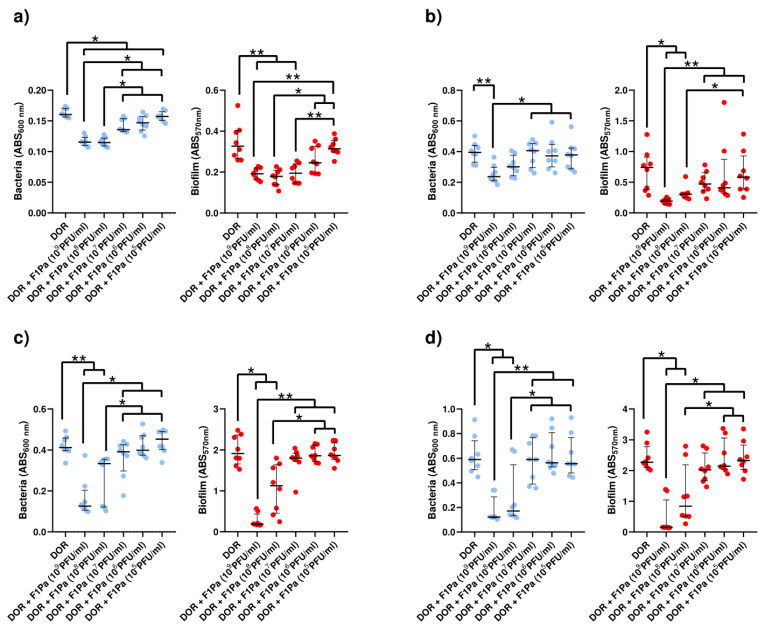
Effect of combined treatment with F1Pa and doripenem (23 µg/mL) on PAO1 (**a**), PA24 (**b**), PA35 (**c**), and PA36 (**d**) in both planktonic bacteria originating from the biofilm (**left**) and biofilm (**right**). The bars represent the median and the interquartile range. * *p*-value < 0.05, **: *p*-value < 0.01 for Dunn’s test pairwise.

The concentration of PAO1 planktonic bacteria derived from biofilm was decreased by 28%, 28%, 15%, and 8% in the presence of 10^9^, 10^8^, 10^7^, and 10^6^ PFU/mL F1Pa, respectively, combined with DOR (23 µg/mL) (*p*-value < 0.05) at 24 h. The concentration of planktonic bacteria derived from biofilm and the concentration of the bacteriophage combined with DOR showed a very strong negative correlation (ρ = −0.8652, *p*-value < 0.0001) at 24 h. Concentrations of 10^9^, 10^8^, and 10^7^ PFU/mL F1Pa combined with DOR were able to reduce the amount of PAO1 biofilm by 41%, 45%, and 40% respectively, (*p*-value < 0.05) at 24 h. The amount of biofilm and the concentration of the bacteriophage combined with DOR showed a strong negative correlation (ρ = −0.7555, *p*-value < 0.0001) at 24 h (Figure 6a).

Only 10^9^ PFU/mL F1Pa combined with DOR was able to decrease by 40% the concentration of PA24 clinical isolate planktonic bacteria derived from biofilm (*p*-value < 0.01) at 24 h. The concentration of planktonic bacteria derived from biofilm and the concentration of the bacteriophage combined with DOR showed a moderate negative correlation (ρ = −0.5160, *p*-value < 0.0001) at 24 h. Concentrations of 10^9^ and 10^8^ PFU/mL F1Pa combined with DOR were able to reduce by 74% and 59% the amount of PÂ24 biofilm, respectively, (*p*-value < 0.05) at 24 h. The amount of biofilm and the concentration of the bacteriophage combined with DOR showed a strong negative correlation (ρ = −0.6789, *p*-value < 0.0001) at 24 h (Figure 6b).

The concentration of PA35 clinical isolate planktonic bacteria derived from biofilm decreased by 69% and 19% in the presence of 10^9^ and 10^8^ PFU/mL F1Pa, respectively, combined with DOR (*p*-value < 0.01) at 24 h. The concentration of planktonic bacteria derived from biofilm and the concentration of the bacteriophage combined with DOR showed a strong negative correlation (ρ = −0.7133, *p*-value < 0.0001) at 24 h. Concentrations of 10^9^ and 10^8^ PFU/mL F1Pa combined with DOR were able to reduce by 90% and 41% the amount of PA35 biofilm, respectively, (*p*-value < 0.05) at 24 h. The amount of biofilm and the concentration of the bacteriophage combined with DOR showed a strong negative correlation (ρ = −0.7151, *p*-value < 0.0001) at 24 h (Figure 6c).

The concentration of PA36 clinical isolate planktonic bacteria derived from biofilm decreased by 79% and 71% in the presence of 10^9^ and 10^8^ PFU/mL F1Pa combined with DOR (*p*-value < 0.05) at 24 h. The concentration of planktonic bacteria derived from biofilm and the concentration of the bacteriophage combined with DOR showed a strong negative correlation (ρ = −0.6076, *p*-value < 0.0001) at 24 h. Concentrations of 10^9^ and 10^8^ PFU/mL F1Pa combined with DOR were able to reduce by 93% and 63% the amount of PA36 biofilm, respectively, (*p*-value < 0.05) at 24 h. The amount of biofilm and the concentration of the bacteriophage combined with DOR showed a strong negative correlation (ρ = −0.6794, *p*-value < 0.0001) at 24 h (Figure 6d).

#### 2.4.4. Imipenem-F1Pa

##### Imipenem (21 µg/mL)-F1Pa

**Figure 7:**
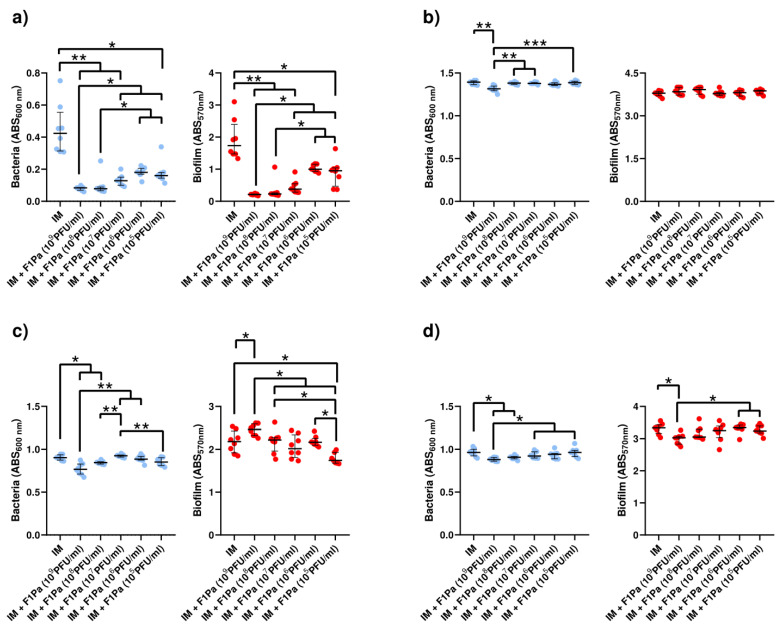
Effect of combined treatment with F1Pa and imipenem (21 µg/mL) on PAO1 (**a**), PA24 (**b**), PA35 (**c**), and PA36 (**d**) in both planktonic bacteria originating from the biofilm (**left**) and biofilm (**right**). The bars represent the median and the interquartile range. * *p*-value < 0.05, **: *p*-value < 0.01, ***: *p*-value < 0.001 for Dunn’s test pairwise.

The concentration of PAO1 planktonic bacteria derived from biofilm decreased by 80%, 81%, 70%, and 62% in the presence of 10^9^, 10^8^, 10^7^_,_ and 10^5^ PFU/mL F1Pa, respectively, combined with IM (21 µg/mL) (*p*-value < 0.05) at 24 h. The concentration of planktonic bacteria derived from biofilm and the concentration of the bacteriophage combined with IM showed a very strong negative correlation (ρ = −0.8392, *p*-value < 0.0001) at 24 h. Concentrations of 10^9^, 10^8^_,_ and 10^7^ PFU/mL F1Pa combined with IM were able to reduce by 88%, 87%, and 78% the amount of PAO1 biofilm, respectively, (*p*-value < 0.05) at 24 h. The amount of biofilm and the concentration of the bacteriophage combined with IM showed a very strong negative correlation (ρ = −0.8792, *p*-value < 0.0001) at 24 h (Figure 7a).

Only 10^9^ PFU/mL F1Pa combined with IM was able to decrease by 6% the concentration of PA24 clinical isolate planktonic bacteria derived from biofilm (*p*-value < 0.05) at 24 h. The concentration of planktonic bacteria derived from biofilm and the concentration of the bacteriophage combined with IM showed a moderate negative correlation (ρ = −0.4729, *p*-value < 0.0001) at 24 h. No concentration of the bacteriophage combined with IM was able to reduce the amount of PA24 biofilm at 24 h. There was no correlation between the amount of PA24 biofilm and the concentration of the bacteriophage combined with IM (*p*-value = 0.1355) at 24 h (Figure 7b).

The concentration of PA35 clinical isolate planktonic bacteria derived from biofilm decreased in the presence of 10^9^ and 10^8^ PFU/mL F1Pa combined with IM by 15% and 6%, respectively, (*p*-value < 0.05) at 24 h. The concentration of planktonic bacteria derived from biofilm and the concentration of the bacteriophage combined with IM showed a moderate negative correlation (ρ = −0.4359, *p*-value < 0.0001) at 24 h. Only 10^9^ PFU/mL F1Pa combined with IM was able to reduce the amount of PA35 biofilm by 13% (*p*-value < 0.05) at 24 h. The amount of PA35 biofilm and the concentration of the bacteriophage combined with IM showed moderate negative correlation (ρ = −0.4376, *p*-value < 0.0001) at 24 h (Figure 7c).

The concentration of PA36 clinical isolate planktonic bacteria derived from biofilm decreased by 9% and 6% in the presence of 10^9^ and 10^8^ PFU/mL F1Pa, respectively, combined with IM (*p*-value < 0.05) at 24 h. The concentration of planktonic bacteria derived from biofilm and the concentration of the bacteriophage combined with IM showed a strong negative correlation (ρ = −0.6045, *p*-value < 0.0001) at 24 h. Only 10^9^ PFU/mL F1Pa combined with IM was able to reduce the amount of PA36 biofilm by 9% (*p*-value < 0.05) at 24 h. The amount of PA36 biofilm and the concentration of the bacteriophage combined with IM showed moderate negative correlation (ρ = −0.4605, *p*-value < 0.0001) at 24 h (Figure 7d).

##### Imipenem (39 µg/mL)–F1Pa

**Figure 8:**
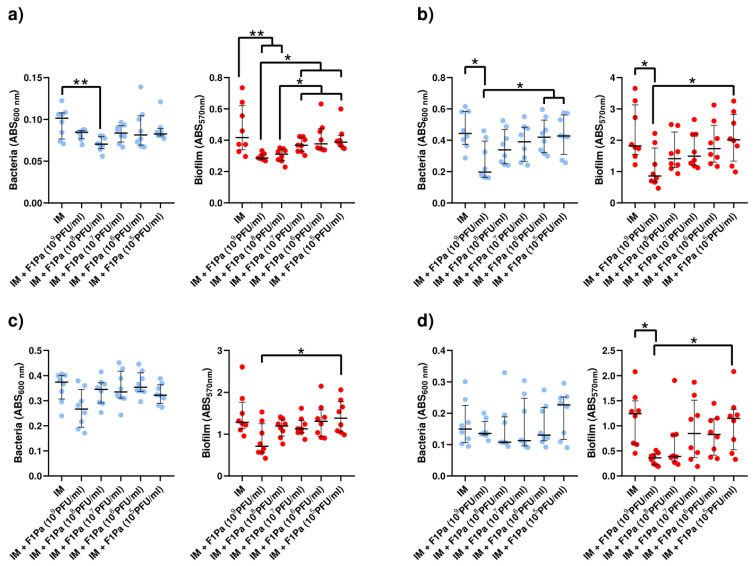
Effect of combined treatment with F1Pa and imipenem (39 µg/mL) on PAO1 (**a**), PA24 (**b**), PA35 (**c**), and PA36 (**d**) in both planktonic bacteria originating from the biofilm (**left**) and biofilm (**right**). The bars represent the median and the interquartile range. * *p*-value < 0.05, **: *p*-value < 0.01 for Dunn’s test pairwise.

Only 10^8^ PFU/mL F1Pa combined with IM (39 µg/mL) was able to decrease the concentration of PAO1 planktonic bacteria derived from biofilm by 31% (*p*-value < 0.05) at 24 h. The concentration of planktonic bacteria derived from biofilm and the concentration of the bacteriophage combined with IM showed a weak negative correlation (ρ = −0.3465, *p*-value < 0.05) at 24 h. Concentrations of 10^9^ and 10^8^ PFU/mL F1Pa combined with IM were able to reduce by 31% and 25% the amount of PAO1 biofilm, respectively, (*p*-value < 0.01) at 24 h. The amount of PAO1 biofilm and the concentration of the bacteriophage combined with IM showed strong negative correlation (ρ = −0.6776, *p*-value < 0.0001) at 24 h (Figure 8a).

Only 10^9^ PFU/mL F1Pa combined with IM was able to decrease the concentration of PA24 planktonic bacteria derived from biofilm by 56% (*p*-value < 0.05) at 24 h. The concentration of planktonic bacteria derived from biofilm and the concentration of the bacteriophage combined with IM showed a moderate negative correlation (ρ = −0.4737, *p*-value < 0.05) at 24 h. In addition, only 10^9^ PFU/mL F1Pa combined with IM was able to reduce the amount of PA24 biofilm by 53% (*p*-value < 0.05) at 24 h. The amount of PA24 biofilm and the concentration of the bacteriophage combined with IM showed moderate negative correlation (ρ = −0.4508, *p*-value < 0.0001) at 24 h (Figure 8b).

No concentration of F1Pa combined with IM was able to decrease the concentration of PA35 clinical isolate planktonic bacteria derived from biofilm at 24 h. The concentration of planktonic bacteria derived from biofilm and the concentration of the bacteriophage combined with IM showed a weak negative correlation (ρ = −0.2871, *p*-value < 0.05) at 24 h. Furthermore, no concentration of bacteriophage combined with AZ was able to reduce the amount of PA35 biofilm at 24 h. The amount of biofilm and the concentration of the bacteriophage combined with IM showed a moderate negative correlation (ρ = −0.4253, *p*-value < 0.05) at 24 h (Figure 8c).

No concentration of F1Pa combined with IM was able to decrease the concentration of PA36 clinical isolate planktonic bacteria derived from biofilm at 24 h. There was no correlation between the concentration of planktonic bacteria derived from biofilm and the concentration of the bacteriophage combined with IM (*p*-value = 0.3518) at 24 h. Only 10^9^ PFU/mL F1Pa combined with IM was able to reduce the amount of PA36 biofilm by 29% (*p*-value < 0.01) at 24 h. The amount of biofilm and the concentration of the bacteriophage combined with IM showed a moderate negative correlation (ρ = −0.5301, *p*-value < 0.05) at 24 h (Figure 8d).

#### 2.4.5. Meropenem–F1Pa

**Figure 9:**
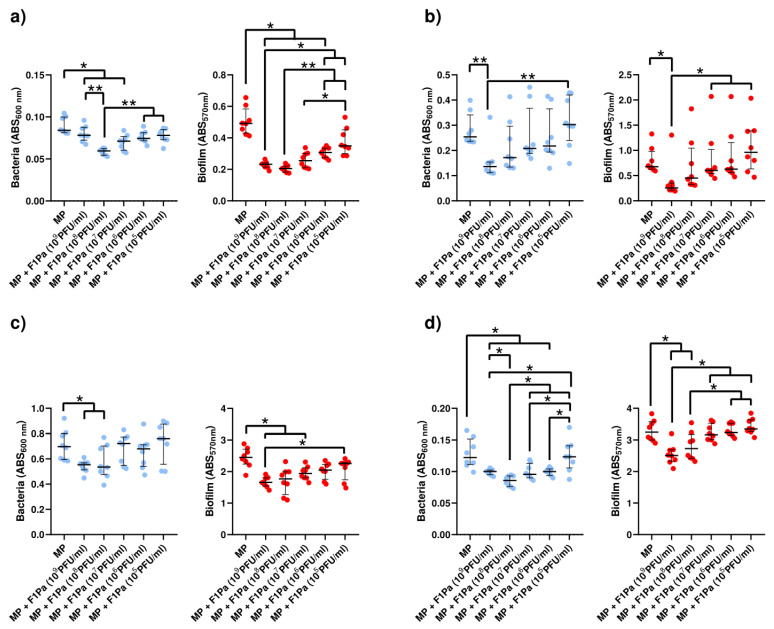
Effect of combined treatment with F1Pa and meropenem (34.3 µg/mL) on PAO1 (**a**), PA24 (**b**), PA35 (**c**), and PA36 (**d**) in both planktonic bacteria originating from the biofilm (**left**) and biofilm (**right**). The bars represent the median and the interquartile range. * *p*-value < 0.05, **: *p*-value < 0.01 for Dunn’s test pairwise.

The concentration of PAO1 planktonic bacteria derived from biofilm decreased in the presence of 10^8^ and 10^7^ PFU/mL F1Pa combined with MP (34.3 µg/mL) by 29% and 15%, respectively, (*p*-value < 0.01) at 24 h. The concentration of planktonic bacteria derived from biofilm and the concentration of the bacteriophage combined with MP showed a moderate negative correlation (ρ = −0.4323, *p*-value < 0.0001) at 24 h. Concentrations of 10^9^, 10^8^, 10^7^, and 10^6^ PFU/mL F1Pa combined with MP were able to reduce by 53%, 58%, 48%, and 37% the amount of PAO1 biofilm, respectively, (*p*-value < 0.01) at 24 h. The amount of biofilm and the concentration of the bacteriophage combined with MP showed a very strong negative correlation (ρ = −0.8458, *p*-value < 0.0001) at 24 h (Figure 9a).

Only 10^9^ PFU/mL F1Pa combined with MP was able to decrease the concentration of PA24 clinical isolate planktonic bacteria derived from biofilm by 62% (*p*-value < 0.01) at 24 h. The concentration of planktonic bacteria derived from biofilm and the concentration of the bacteriophage combined with MP showed a moderate negative correlation (ρ = −0.5274, *p*-value < 0.0001) at 24 h. In addition, only 10^9^ PFU/mL F1Pa combined with MP was able to reduce the amount of PA24 biofilm by 47% (*p*-value < 0.01) at 24 h. The amount of PA24 biofilm and the concentration of the bacteriophage combined with MP showed a moderate negative correlation (ρ = −0.5116, *p*-value < 0.0001) at 24 h (Figure 9b).

The concentration of PA35 clinical isolate planktonic bacteria derived from biofilm decreased in the presence of 10^9^ and 10^8^ PFU/mL F1Pa combined with MP by 21% and 23%, respectively, (*p*-value < 0.05) at 24 h. The concentration of planktonic bacteria derived from biofilm and the concentration of the bacteriophage combined with MP showed a moderate negative correlation (ρ = −0.4667, *p*-value < 0.0001) at 24 h. Concentrations of 10^9^, 10^8^, and 10^7^ PFU/mL F1Pa combined with MP were able to reduce the amount of PA35 biofilm by 32%, 28%, and 21%, respectively, (*p*-value < 0.05) at 24 h. The amount of PA35 biofilm and the concentration of the bacteriophage combined with MP showed a strong negative correlation (ρ = −0.6498, *p*-value < 0.0001) at 24 h (Figure 9c).

The concentration of PA36 clinical isolate planktonic bacteria derived from biofilm decreased in the presence of 10^9^, 10^8^, 10^7^, and 10^6^ PFU/mL F1Pa combined with MP by 18%, 30%, 22%, and 18%, respectively, (*p*-value < 0.05) at 24 h. The concentration of planktonic bacteria derived from biofilm and the concentration of the bacteriophage combined with MP showed a moderate negative correlation (ρ = −0.5874, *p*-value < 0.0001) at 24 h. Concentrations of 10^9^ and 10^8^ PFU/mL F1Pa combined with MP were able to reduce by 23% and 16%, respectively, the amount of PA36 biofilm (*p*-value < 0.05) at 24 h. The amount of PA36 biofilm and the concentration of the bacteriophage combined with MP showed a moderate negative correlation (ρ = −0.5922, *p*-value < 0.0001) at 24 h (Figure 9d).

#### 2.4.6. Piperacillin/Tazobactam–F1Pa

**Figure 10:**
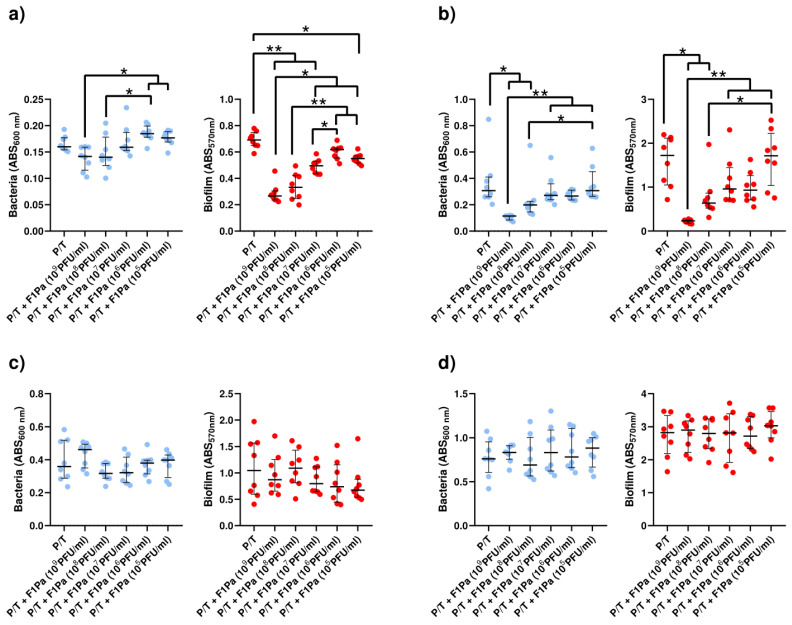
Effect of combined treatment with F1Pa and piperacillin/tazobactam (64.3 µg/mL) on PAO1 (**a**), PA24 (**b**), PA35 (**c**), and PA36 (**d**) in both planktonic bacteria originating from the biofilm (left) and biofilm (right). The bars represent the median and the interquartile range. * *p*-value < 0.05, **: *p*-value < 0.01 for Dunn’s test pairwise.

No concentration of F1Pa combined with P/T (64.3 µg/mL) decreased the concentration of PAO1 planktonic bacteria derived from biofilm at 24 h. The concentration of planktonic bacteria stemming from biofilm and the concentration of the bacteriophage combined with P/T showed a moderate negative correlation (ρ = −0.4279, *p*-value < 0.01). Concentrations of 10^9^, 10^8^, 10^7^, and 10^5^ PFU/mL F1Pa combined with P/T were able to reduce by 62%, 52%, 28%, and 20% the amount of PAO1 biofilm, respectively, (*p*-value < 0.05). The amount of PAO1 biofilm and the concentration of the bacteriophage combined with P/T showed a very strong negative correlation (ρ = −0.8687, *p*-value < 0.0001) (Figure 10a).

The concentration of PA24 clinical isolate planktonic bacteria originating from biofilm decreased in the presence of 10^9^ and 10^8^ PFU/mL F1Pa combined with P/T by 63% and 35%, respectively, (*p*-value < 0.05). The concentration of planktonic bacteria stemming from biofilm and the concentration of the bacteriophage combined with P/T showed a strong negative correlation (ρ = −0.6948, *p*-value < 0.0001). Concentrations of 10^9^ and 10^8^ PFU/mL F1Pa combined with P/T reduced the amount of PA24 biofilm by 87% and 63%, respectively (*p*-value < 0.05). The amount of PA24 biofilm and the concentration of the bacteriophage combined with P/T showed a strong negative correlation (ρ = −0.7344, *p*-value < 0.0001) (Figure 10b).

No concentration of F1Pa combined with P/T decreased the concentration of PA35 clinical isolate planktonic bacteria from the biofilm. There was no correlation between the concentration of PA35 clinical isolate in the planktonic state sourced from biofilm and the concentration of the bacteriophage combined with P/T (*p*-value = 0.9243). In addition, no concentration of F1Pa combined with P/T was able to reduce the amount of PA35 biofilm. There was no correlation between the amount of PA35 biofilm and the concentration of the bacteriophage combined with P/T (*p*-value = 0.4106) (Figure 10c).

No concentration of F1Pa combined with P/T decreased the concentration of PA36 clinical isolate planktonic bacteria derived from biofilm. There was no correlation between the concentration of PA36 clinical isolate in planktonic state stemming from biofilm and the concentration of the bacteriophage combined with P/T (*p*-value = 0.9361). Furthermore, no concentration of F1Pa combined with P/T was able to reduce the amount of PA36 biofilm. There was no correlation between the amount of PA36 biofilm and the concentration of the bacteriophage combined with P/T (*p*-value = 0.6258) (Figure 10d).

## 3. Discussion

Infections of the dermis (including burns, surgical-site infections, and non-healing diabetic foot ulcers) have an enormous impact on healthcare. One of the main pathogens in burns [58], diabetic foot ulcers [59], and chronic wounds [60,61] is *P. aeruginosa*. Our objective was to develop a biofilm model that closely resembled the kind of biofilm that we could observe in chronic wounds in clinical practice. The developed model uses red blood cells and plasma to directly supply these elements in support of our goals to replicate the nutritional environment more accurately in a chronic wound. In addition, bovine plasma and *S. aureus* coagulase enzyme (converts fibrinogen to fibrin) were found to be critical for the biofilm growth and anatomy of the wound-like medium, respectively [62]. An evaluation was undertaken of how the biofilm formation ability of the *P. aeruginosa* bacteria population was affected by bacteriophage treatment. The wound-like medium was exposed to the maximum concentration of F1Pa to evaluate the bacteria population responses. As shown in the results, F1Pa was able to inhibit the biofilm formation in the wound-like medium of *P. aeruginosa* reference strain PAO1 and clinical isolate PA24; the initial inoculum used was 5 × 10^5^ CFU/mL, and the final bacterial concentrations were near 3.2 × 10^5^ and 1 × 10^5^ CFU/mL, respectively. Meanwhile, the bacteriophage only was able to inhibit bacterial growth in the PA35 and PA36 clinical isolates, as the final bacterial concentrations were 1.6 × 10^6^ and 6.3 × 10^6^. Phage therapy in in vitro wound models was also described by [63], whose authors designed a new, combined bacteriophage–antibiotic therapy using phages targeting *P. aeruginosa* and *S. aureus* in combination with gentamicin as an adjuvant in a wound-like medium.

Combination therapies with additional antimicrobial agents are a wise clinical choice because each agent used in monotherapy with a particular target may encounter resistance in *P. aeruginosa*. The potential synergistic activity of the bacteriophage F1Pa in combination with several β-lactam antibiotics against *P. aeruginosa* was investigated in the present manuscript. The transpeptidase that is involved in cross-linking peptides to generate peptidoglycan is acylated by beta-lactam antibiotics, which prevents the final step in peptidoglycan formation. Penicillin-binding proteins are targets of these antibiotics. By interfering with the terminal transpeptidation pathway, this binding causes the bacterial cell to undergo autolysis, which further results in lysis and loss of viability. Beneficial interactions between the bacteriophage F1Pa and beta-lactam antibiotics were shown, in both planktonic and biofilm forms, and no signs of antagonism were seen with *P. aeruginosa* clinical isolates. These results are consistent with those observed in other studies with beta-lactam antibiotics, specifically meropenem [55,56].

Examining the individual growth curves from our phage–antibiotic studies, it can be observed that the addition of the bacteriophage F1Pa to doripenem led to better killing at late time points. These results are consistent with the studies described previously [64] on the combination of phages and beta-lactam antibiotics. Lusiak-Szelachowska et al. [65] suggested six mechanisms to explain the phenomenon of phage–antibiotic synergy (PAS). The total inhibition of the bacterial growth of the three clinical strains adding together doripenem and MOI 10 F1Pa compared with the partial inhibition of doripenem or F1Pa alone (the last one published previously [54]) suggests that the main mechanism involved in this synergy could be increased antibiotic susceptibility due to the presence of the phage, as phage-resistant bacteria may contain mutations that resensitize the strains to beta-lactams. To understand the differences encountered with different MOIS, it is important to recall that at an MOI of 1, only 33% of bacteria are infected during the first cycle, while at an MOI of 10, more than 95% of bacteria are affected (Poisson’s law).

Regarding the phage–antibiotic inhibition biofilm assays, the determinations of bacterial growth (Abs _600nm_) and MTT (Abs _570nm_) allow us to determine the concentration of bacteria in the planktonic state derived from the preformed biofilm and the viability of bacteria belonging to the preformed biofilm, respectively. As can be observed, the combination of aztreonam and the F1Pa phage diminished the production of planktonic bacteria of the *P. aeruginosa* reference strain PAO1 and the clinical PA24 and PA36 strains. The phage–aztreonam combination also reduced the biofilm viability of PAO1 and PA24. However, neither the concentration of the bacteria PA35 in the planktonic state nor the viability of the PA35 biofilm was affected by the phage–aztreonam combination due to its high resistance (MIC aztreonam PA35 > 64 mg/mL). Remarkably, the bacteriophage–carbapenem combination inhibited the production of planktonic bacteria and reduced the biofilm viability of the *P. aeruginosa* reference and clinical strains. The combination of piperacillin/tazobactam with the bacteriophage reduced the production of planktonic bacteria and the viability of *P. aeruginosa* biofilm in the reference strain PAO1 and the PA24 clinical isolate, but not in the PA35 (MIC = 128 mg/mL) or PA36 (MIC = 64 mg/mL) clinical strains due to their high resistance to piperacillin/tazobactam. Although they are excellent bactericides, beta-lactam antibiotics are not very effective against biofilms. Therefore, they have a high bactericidal effect by attacking the bacteria left in a planktonic state from the biofilm’s disintegration by the phage F1Pa.

Bacteriophage–antibiotic combinations were previously reported as a promising therapeutic strategy against *P. aeruginosa* biofilm [49,50,55,56,64,66,67,68,69], including beta-lactam antibiotics such as ceftazidime [50,56,67], cefepime [64], and meropenem [49,55,56,64].

## 4. Material and Methods

### 4.1. Bacterial Strains and Growth Conditions

The American Type Culture Collection (ATCC) (Manassas, VA, USA) provided the reference strain of *P. aeruginosa*, ATCC15692 (PAO1). The Department of Microbiology of the Hospital Universitario Fundación Jiménez Díaz (HUFJD) submitted three *P. aeruginosa* clinical isolates of patients (Table 2). MALDI-TOF (Bruker, Preston, VIC, Australia) was used to identify clinical isolates of *P. aeruginosa*. The clinical isolates of *P. aeruginosa* and PAO1 were kept at −80 °C in Difco^TM^ skimmed milk (East Rutherford, NJ, USA). The clinical isolates were cultured in tryptic soy broth (TSB) (BioMérieux, Marcy-l’Étoile, France) and plated from frozen skimmed milk stocks onto tryptic soy agar with 5% sheep blood (TSS) plates (BioMérieux, France).

### 4.2. Bacteriophage Isolation

Samples of wastewater were collected from the HUFJD sewerage tubes, which receive feces. Fifty-mL samples were centrifuged for 10 min at 4500 rpm to remove feces and cell debris. To eliminate germs and debris, a 0.22 µm filter was used to filter the supernatant. Using the double-layer agar method [70], 100 µL of the filtered solution, 100 µL of the PAO1 overnight culture, and 3 mL of molten 0.2% (*w/v*) LB agar (Invitrogen, Waltham, MA, USA) (LBA) were combined and plated on 1.5% (*w/v*) LBA plates. Plaques that developed after the incubation period of one night indicated the existence of the phage. Using a plastic Pasteur pipette, one plaque was selected and put into a 1.5 mL microcentrifuge tube. This tube held 1 mL of sodium magnesium buffer (SM); this contained 10 mM MgSO_4_ (Thermo Fisher Scientific, Waltham, MA, USA); 10 mM CaCl_2_ (Thermo Fisher Scientific, Waltham, MA, USA) and 50 mM Tris HCl (Sigma Aldrich, Merck, Darmstadt, Germany), pH 7.5. The mixture was then vigorously vortexed for 5 min and centrifuged at 4000 g for five minutes, and the supernatant was stored at 4 °C.

### 4.3. Bacteriophage Propagation

The isolated phage was amplified and purified using a two-step propagation process. One hundred µL of PAO1 overnight culture and 100 µL of phage were added to 10 mL of TSB containing 10 mM MgSO_4_ and 10 mM CaCl_2_, and the mixture was incubated overnight at 37 °C with 200 rpm shaking for small-scale phage amplification. After centrifugation (4500 rpm, 10 min), the phage-containing supernatant was harvested, and bacterial debris was removed by filtration (0.22 µm PES syringe filter). Phage titration was used to determine the phage count.

To amplify the phage on a wide scale, 500 µL of the PAO1 overnight culture was incubated for 20 min with 50 mL of TSB. Following the incubation period, 100 µL of phage was added, along with MgSO_4_ and CaCl_2_ cations, to reach a final concentration of 10 mM. The coculture was then incubated for an additional night at 37 °C with 200 rpm shaking. On a modest scale, the phage lysis data were extracted as previously mentioned.

### 4.4. Bacteriophage Titration

The double-layer agar method [71] was used to determine the phage titer. In summary, 3 mL of melted 0.2% (*w/v*) LBA was mixed with 100 µL of the PAO1 overnight broth culture, and the mixture was then spread onto a 1.5% (*w/v*) LBA plate. In SM buffer, bacteriophages were serially diluted. Overnight, the plates were incubated at 37 °C. The calves of serial dilutions were counted to determine the phage titer.

### 4.5. In Vitro Wound-like Medium

The composition of the wound-like medium (WLM) consisted of 45% Bolton broth (Sigma Aldrich) enhanced with 10 mM CaCl_2_ and 10 mM MgSO_4_ and pre-bred Bolton broth with *Staphylococcus aureus* ATCC 29213; 50% rabbit plasma diluted in human serum; and 5% laked horse red blood cells (RBC) as described previously [62]. A 0.5 mL volume of WLM was placed in flat-bottom, 24-well cell culture plate (Thermo Fisher Scientific, Waltham, MA, USA) and incubated at 37 °C and 5% CO_2_ for 24 h. After incubation, we inoculated 50 µL per well of different concentrations of bacteriophage in saline solution supplemented with 10 mM CaCl_2_ and 10 mM MgSO_4_, except for the positive control. Thereafter, 5 µL of saline solution containing 10^8^ CFU/mL of bacteria was added before incubation at 37 °C and 5% CO_2_ for 6 h. The inhibition of wound biofilm formation was determined by the drop plate method.

### 4.6. Bacteriophage–Antibiotic Interactions

Phage–antibiotic interactions were examined in a MicroWell^TM^ flat-bottom 96-well plate (Thermo Fisher Scientific, Waltham, MA, USA) using a modified checkerboard method [72]. Antibiotic concentrations in columns were varied twofold on each plate (from 2 to 12), with tenfold concentrations of the bacteriophage in rows (from B to F). Column A contained only the bacteriophage (to obtain the MIC of the phage), while row A only contained the antibiotic (to obtain the MIC of the antibiotic). The A1 well contained the control of bacterial growth. We inoculated 100 µL of MHB supplemented with 10 mM CaCl_2_, and 10 mM MgSO_4_ containing 10^6^ CFU/mL of bacteria, 50 µL with different concentrations of F1PA in supplemented MHB, and 50 µL with different concentrations of the antibiotic in supplemented MHB per well. The sole substance in the positive control well was broth, which was 200 µL of MHB supplemented with 10^6^ CFU/mL of bacteria. For at least 18 h, the plate was incubated at 37 °C in a wet chamber. Pseudomonal growth was assessed following incubation by monitoring the absorbance at 595 nm. This experiment was performed in triplicate.

The fractional inhibitory concentration (FIC) index was used to quantify the interaction between each antibiotic and the bacteriophage. Regarding two antimicrobial substances, A and B, functioning separately or jointly:

$$\mathrm{FIC}\;\mathrm{Index}=\frac{A}{MICA}+\frac{B}{MICB}$$

where the MIC values of the bacteriophage and the antibiotic individually are represented by A and B, respectively. The MIC of the antibiotic in combination with the bacteriophage is denoted by MICA, while the MIC of the bacteriophage in conjunction with the antibiotic is denoted by MICB. The FIC index is the sum of FICA and FICB. An FICI ≤ 0.50 points out synergism, an FICI between 0.50 and 1.00 represents an additive effect, an FICI from 1.00 to 2.00 was defined as indifference, and an FICI greater than 2.00 represents antagonism [57].

### 4.7. Phage–Antibiotic Inhibition Assays

Bacteriophage–antibiotic interaction was assessed at different concentrations of the phage and a single concentration of the antibiotic using inhibition assays in liquid. The antibiotic used was doripenem (Sigma Aldrich, Merck, Darmstadt, Germany) at plasma median peak concentration [73]. In brief, an inoculation of each clinical bacterium (10^9^ CFU/mL) was prepared, and the required volumes of the bacteriophage and the antibiotic stock solution were added to achieve the different concentrations of bacteriophage F1PA (MOI 10 and MOI 1) and doripenem (8.79 µg/mL), except for the antibiotic control without the phage and the positive growth control with neither the phage nor the antibiotic (n = 10 per combination). The samples were incubated at 37 °C with a shaking orbital amplitude of 5 mm. Every 5 min for 48 h, the OD_595_ value was measured. This experiment was performed in duplicate.

### 4.8. Phage–Antibiotic Effect on Pseudomonal Biofilm

The bacteriophage–antibiotic effect on pseudomonal biofilm was determined using a methodology previously described [74], with modifications. The antibiotics used were aztreonam, doripenem, imipenem, meropenem, and piperacillin/tazobactam (Sigma Aldrich, Merck, Darmstadt, Germany) at plasma median peak concentration [73,75,76,77,78,79]. Biofilm formation took place on the bottom of a MicroWell^TM^ plate. A 96-well plate was prepared in such a way as to use a specific antibiotic concentration in columns and tenfold concentrations of the bacteriophage in rows, except for the positive control (n = 8 per combination). The lid was placed on the treatment plate, and it was incubated at 37 °C and 5% CO_2_ for 24 h. After incubation, the pseudomonal concentration was determined by measuring the absorbance at 400 nm, and bacterial viability was determined by addition of 20 µL of MTT (5 mg/mL) (Sigma Aldrich, Merck, Darmstadt, Germany), followed by incubation for 1 h at 37 °C, 5% CO_2_ and with shaking at 110 rpm in a wet chamber. Thereafter, we measured the absorbance at 570 nm. This experiment was performed in triplicate.

### 4.9. Statistical Analysis

Everything related to statistical analysis was performed with R (R Core Team, 2017) and the R command-line tool; however, GraphPad Prism v.8 (GraphPad Prism, version 8.0.1 (86); Windows Version by Software MacKiev © 2020–2018 GraphPad Software, LLC.; San Diego, CA, USA) and the STATA statistical software, release 11 (StataCorp, 2009, StataCorp LP., College Station, TX, USA), were used for linear regressions. Statistics such as Shapiro–Wilk or Kolmogorov–Smirnov were used to assess the distribution of the data. For every computed variable, the median and interquartile range (a non-normal distribution) are given as descriptive statistics. Two groups were compared using a non-parametric Mann–Whitney test that took equality of variance into account, and additional groups were compared using a non-parametric Kruskal–Wallis test. By using a Benjamini–Hochberg approach in conjunction with Dunn’s pairwise test, the bacteriophage inhibition of bacterial biofilm was examined. The bacteriophage’s inhibition of biofilm and the combination of the antibiotic and bacteriophage was examined using Dunn’s paired test and the Benjamini–Hochberg method. The correlation between the amount of biofilm and the bacteriophage concentration was determined by Pearson’s correlation coefficient and classified into very weak (0–0.2), weak (0.2–0.4), moderate (0.4–0.6), strong (0.6–0.8), and very strong (0.8–1) [80]. The significance level was established at α = 0.05.

## Figures and Tables

**Table 1:** Fractional inhibitory concentration (FIC) index (FICI). Synergism: FICI ≤ 0.5; additive effect: 0.5 < FICI ≤ 1; indifference: 1 < FICI < 4; antagonism: FICI ≥ 4 [57].

	PAO1	PA24	PA35	PA36
Aztreonam	≤0.60	0.23	0.03	≤0.13
Doripenem	≤0.60	0.10	1.00	≤0.12
Imipenem	≤2.00	0.10	1.01	≤0.10
Meropenem	≤2.00	0.10	0.10	≤0.12
Piperacillin/tazobactam	≤0.60	0.13	1.01	≤0.13

**Table 2:** Clinical isolates of *P. aeruginosa* from HUFJD. Amikacin (AMK), aztreonam (AZ), ceftolozane/tazobactam (C/T), cefepime (CEF), ceftazidime (CFT), ciprofloxacin (CIP), colistin (CO), doripenem (DOR), gentamicin (GE), imipenem (IM), meropenem (MP), piperacillin/tazobactam (P/T), and tobramycin (TOB) are examples of antimicrobial susceptibility (S) and resistance (R) profiles for various antibiotics.

	PA24	PA35	PA36
**Source**	Sputum	-	Sputum
**MDR/XDR**	MDR	XDR	MDR
**AMK**	S	S	S
**AZ**	S	R	S
**C/T**	S	R	S
**CEF**	S	R	S
**CFT**	S	R	R
**CIP**	R	R	R
**CO**	S	S	S
**DOR**	R	R	R
**GE**	R	R	R
**IM**	R	R	R
**MP**	S	R	S
**P/T**	S	R	R
**TOB**	R	R	R

## Data Availability

No new data were created.
